# Supplementary material for: Exchange on dynamic encounter networks allows plant mitochondria to collect complete sets of mitochondrial DNA products despite their incomplete genomes
Source: Quant Plant Biol. 2022 Sep 9;3:e18. doi: 10.1017/qpb.2022.15 (PMC10095876; doi:10.1017/qpb.2022.15)
Supplement: Supplementary file 1 [file S2632882822000157sup001.pdf]

Supplementary Information

Network phrasing of problem

Our network phrasing is as follows. Allow each node to have two labels *DNA* and *gene*. *DNA* is a binary vector of length L describing the set of genetic elements within a node. *gene* is a real-values vector of length L describing the relative concentration of each gene product in the mitochondrion. When a node's *gene*-label contains L nonzero elements, that mitochondrion has been exposed to every gene product in the full set.

Define an *exchange event* between two nodes a and b, connected by an edge, as follows. If DNA exchange is supported, the *DNA* labels of a and b are exchanged (modelling exchange of genetic information). If gene product sharing is supported, the ith element of the *gene* labels for a and b are both updated to the average of those elements prior to exchange (gene product concentrations are averaged across mitochondria).

Between events, gene product levels decrease if their host mitochondrion does not contain the DNA encoding that product, so $gene_i \to \nu gene_i$ if $DNA_i = 0$, where $\nu \leq 1$ is a gene product decay constant.

A given instance of the problem is defined by initial conditions (*DNA* and *gene* labels for each node), descriptions of where DNA and/or gene product exchange is supported, ν, and an adjacency matrix. We are interested in how the *gene*-labels of nodes (the sets of gene products that mitochondria contain) change as the number of exchange events increases. Following the nomenclature in the main text, the bingo score of a node is the proportion of elements in its *gene* labels that exceed a threshold ϵ, and a bingo is scored when this score is 1.

Coupon collector's problem

The CCP generally describes the process of sampling coupons (which are individual members of a set of coupons $\mathcal{L}$) from a certain number n of 'urns' (entities containing coupons) n. In our system, coupons correspond to individual genome regions (members of the full genome), and urns correspond to mitochondria containing these genes (to further draw the analogy between the CCP and the bingo game for mtDNA products, we refer to the visualised glossary in Supplementary Fig. 10). We consider the CCP faced by an individual mitochondrion – a node s in our encounter network. This node begins with its initial gene, and through encounters can draw from each its neighbours (of which there are $deg(s)$). So its total number of draws is $n(s) = deg(s) + 1$, and the number of distinct coupon types to collect is $|\mathcal{L}| = L$.

Study of the CCP has answered many questions about this system – some examples linked to this system appear in Refs. [Flajolet et al., 1992, Adler et al., 2003, Schilling, 2021]. The most central for us is, given n draws, what is the probability of collecting all L coupons? A classical result, outlined below, is that

$$P(s \text{ scores bingo}) = \sum_{j=0}^{L} (-1)^j \binom{L}{j} \left(\frac{L-j}{L} \right)^{n(s)}, \tag{1}$$

The expected number of neighbours required to score a bingo is also easily found (see below) to be

$$E(n(s)|s \text{ scores bingo}) = LH_L, \tag{2}$$

where H_L is the Lth harmonic number.

CCP result outlines

Consider the different patterns of coupons that can be acquired through n draws. There are L^n possible patterns, which we assume all arise with equal probability. We require the probability of obtaining a pattern in which each of the L coupons is present. To get this we use the inclusion-exclusion principle.

We first write down the probability of obtaining a pattern that is compatible with there being an 'alphabet' of L coupons. The probability of a single draw being compatible with an alphabet of L coupons is $L/L = 1$, so we

begin with a probability of $1^n = 1$. We need to deduct the probability of obtaining a pattern that is compatible with there being an alphabet of $L-1$ coupons, because every such pattern cannot feature all L coupons. The probability of an individual draw not obtaining a given coupon l is $(L-1)/L$, so considering each $l \in \mathcal{L}$ we obtain $L \times ((L-1)/L)^n$.

However, we have now over-counted patterns that are compatible with an even smaller alphabet size of $L-2$. So we need to add back the patterns that we have missed. The probability of an individual draw not obtaining either of a given pair of coupons (l_1, l_2) is $(L-2)/L$, so considering each pair of coupons $(l_1, l_2) \in \mathcal{L}$ we have $\binom{L}{2} \times ((L-2)/L)^n$.

However, we have now over-counted patterns that are compatible with an even smaller alphabet size of $L-3$. We thus need to consider triplets of excluded coupons, and so on. The process continues iteratively, alternating between adding and subtracting terms (including and excluding) until we reach L terms. From the above it should be clear that the final form is

$$P(\text{bingo}) = 1 - L\left(\frac{L-1}{L}\right)^n + \binom{L}{2}\left(\frac{L-2}{L}\right)^n - \ldots \quad (3)$$

$$= \sum_{j=0}^{L}(-1)^j\binom{L}{j}\left(\frac{L-j}{L}\right)^n \quad (4)$$

For example, consider $n = L = 3$. Write the 24 three-character strings of length 3 for the set of patterns: AAA, AAB, ... At the first step we include them all. The next step counts all the strings that do not contain A, all those that do not contain B, and all those that do not contain C. Hence, we remove BBC, BCC, and so on – but AAA, BBB, and CCC each get double-counted (once for each coupon they do not contain). The third step recounts all the strings that do not contain A or B, those that do not contain B or C, and those that do not contain A or C, which are exactly those three strings we previously double-counted. As $L = 3$, this is our final step, and we have successfully retained only those strings in which all coupons feature.

The expected number of draws required for a bingo is easier to compute. If we have collected c coupons, the probability of the next draw obtaining an unseen coupon is $(L-c)/L$. Assuming that draws are Bernoulli trials, a geometric distribution describes the behaviour of the system, giving a mean number of draws $L/(L-c)$ required for the next unseen coupon. The expected overall number is then $\sum_{c=0}^{L} L/(L-c) = L(1/L + 1/(L-1) + \ldots + 1/1) = LH_L$.

It is important to note that the above equations assume an equal probability for each draw, as this is the case in the bingo game where L values are initially scattered uniformly across the mitochondria/nodes. The influence of the coupon distribution for the same problem is beyond the scope of this work and is still an open topic of active research. The interested reader is referred to the work of Shilling [Schilling, 2021].

References

[Adler et al., 2003] Adler, I., Oren, S., and Ross, S. M. (2003). The coupon-collector's problem revisited. *Journal of Applied Probability*, 40(2):513–518.

[Flajolet et al., 1992] Flajolet, P., Gardy, D., and Thimonier, L. (1992). Birthday paradox, coupon collectors, caching algorithms and self-organizing search. *Discrete Applied Mathematics*, 39(3):207–229.

[Pons and Latapy, 2006] Pons, P. and Latapy, M. (2006). Computing communities in large networks using random walks. In *J. Graph Algorithms Appl.* Citeseer.

[Schilling, 2021] Schilling, J. (2021). Results and conjectures on the role of the uniform distribution in the coupon collector's problem with group drawings. *Information Processing Letters*, 169:106112.

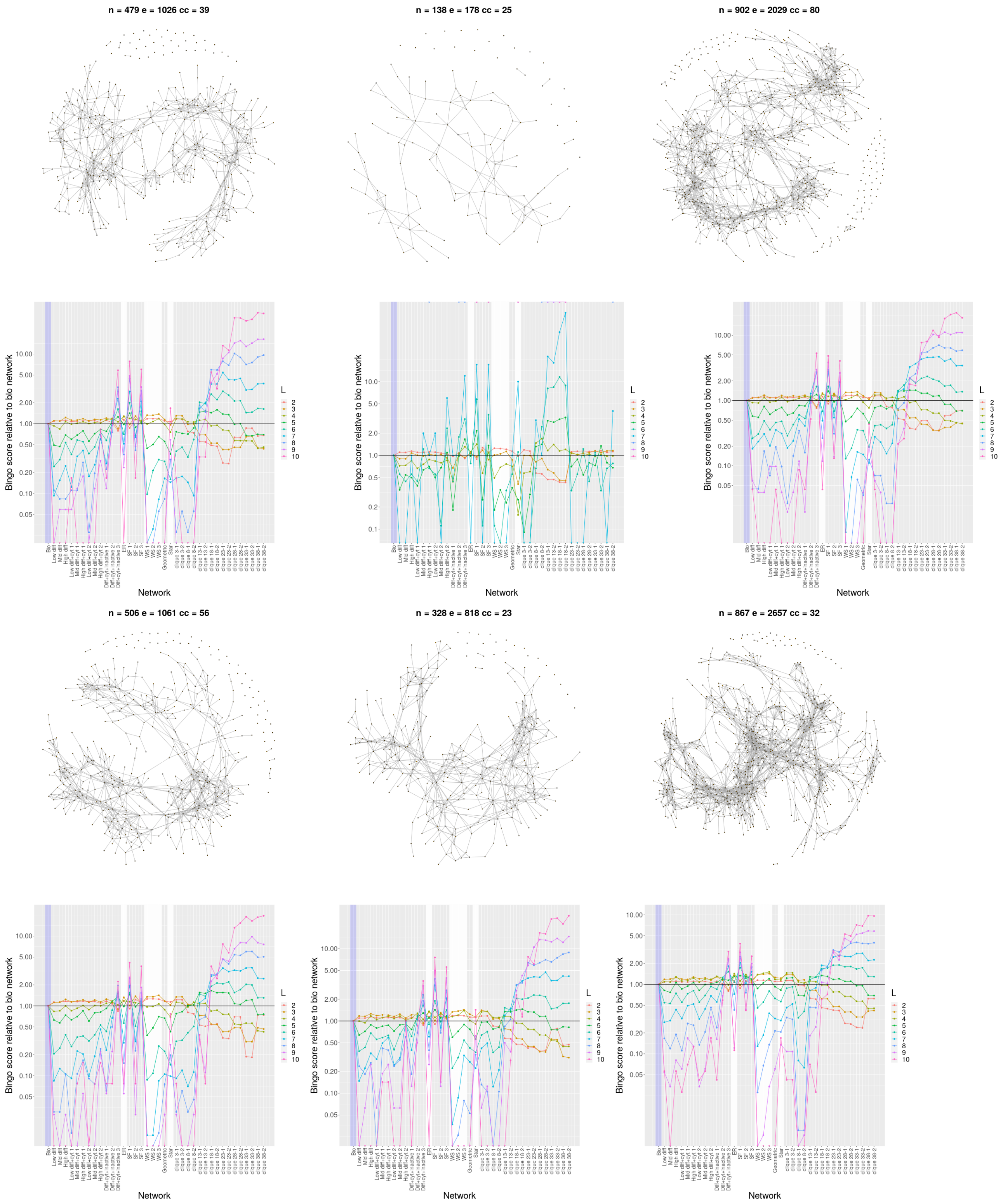

Figure S1: **Comparison of behaviour across different cells.** Examples of encounter network visualisations (n nodes, e edges, cc connected components) and profiles of biological *versus* synthetic partner bingo performance for different single cell observations. All but the top centre cell show very comparable trends. The top centre was unusually small, limiting the size of the mitochondrial population and hence the scale of the encounter network. Correspondingly, the bingo performance for both biological and synthetic partner networks is diminished, especially for high L, but the trends in performance relative to biological networks remain comparable.

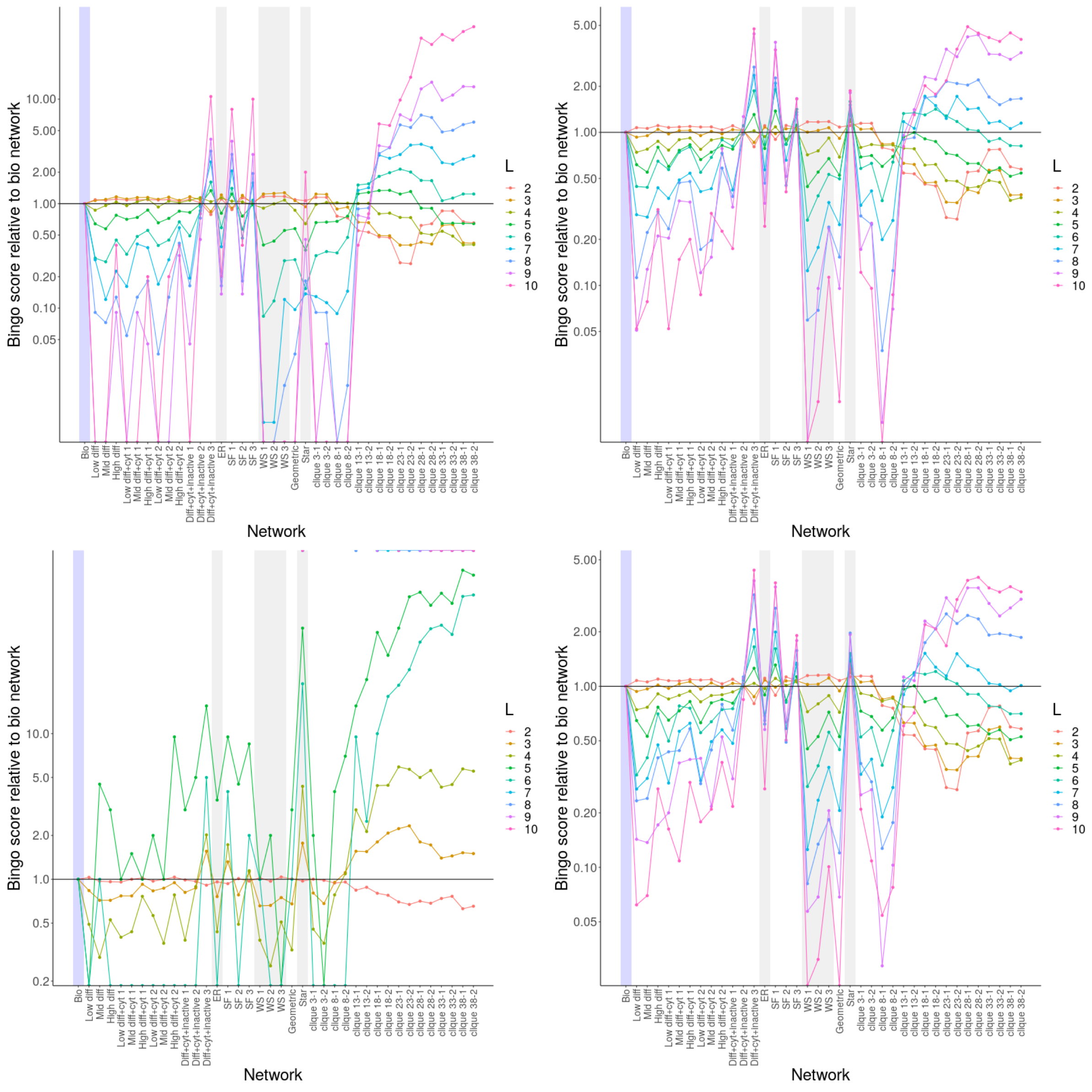

Figure S2: **Bingo performance of different networks under different model structures and initial conditions.** (top left) Empty ICs, (top right) full ICs with intermediate degradation, (bottom left) full ICs with high degradation, (bottom right) full ICs with intermediate degradation and low probability of mtDNA exchange. For high degradation cases, no bingos occur for higher values of *L*.

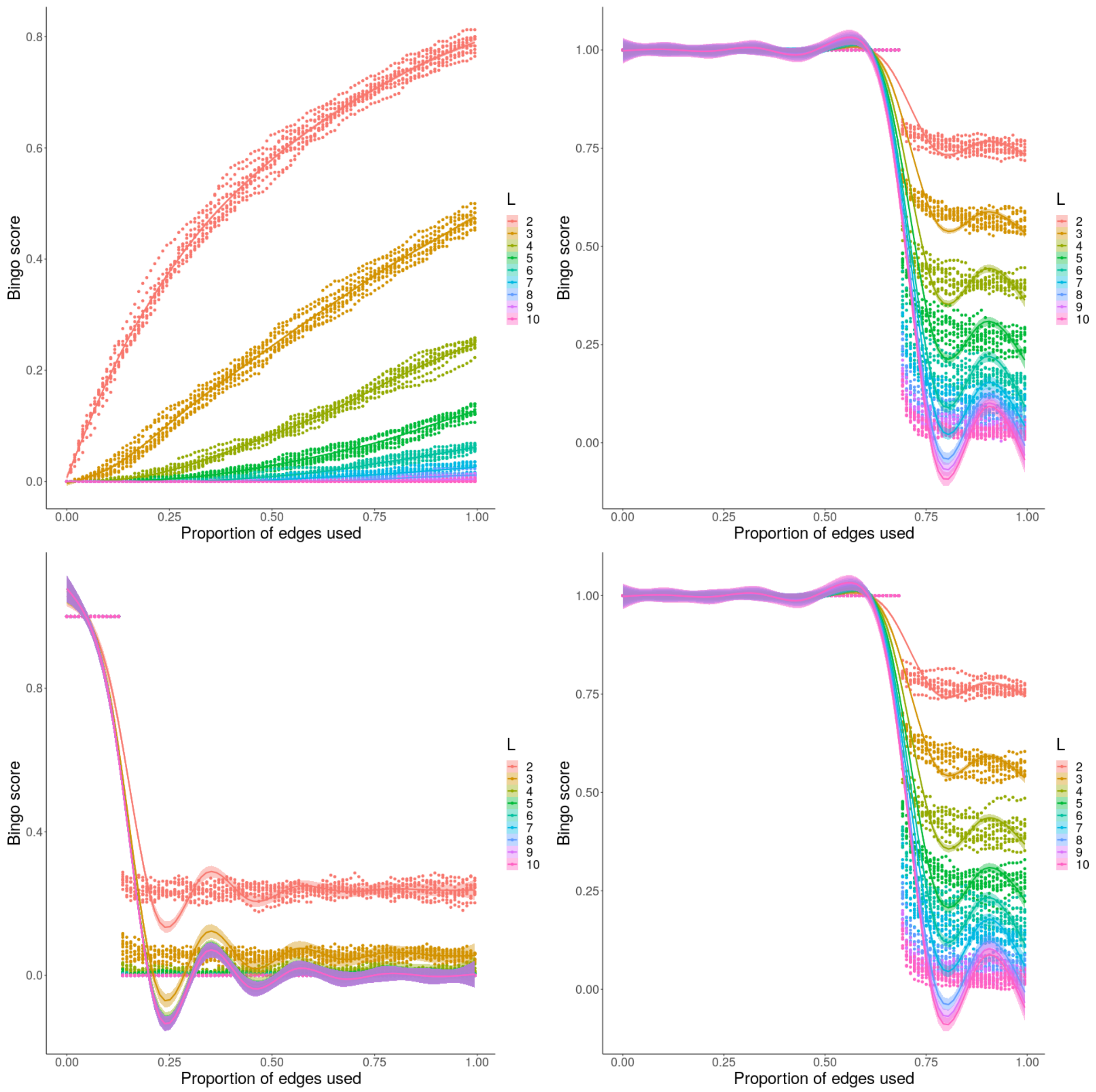

Figure S3: **Dynamics of bingo on biological networks under different model structures and initial conditions.** (top left) Empty ICs, (top right) full ICs with intermediate degradation, (bottom left) full ICs with high degradation, (bottom right) full ICs with intermediate degradation and low probability of mtDNA exchange. For high degradation cases, no bingos occur for higher values of *L*.

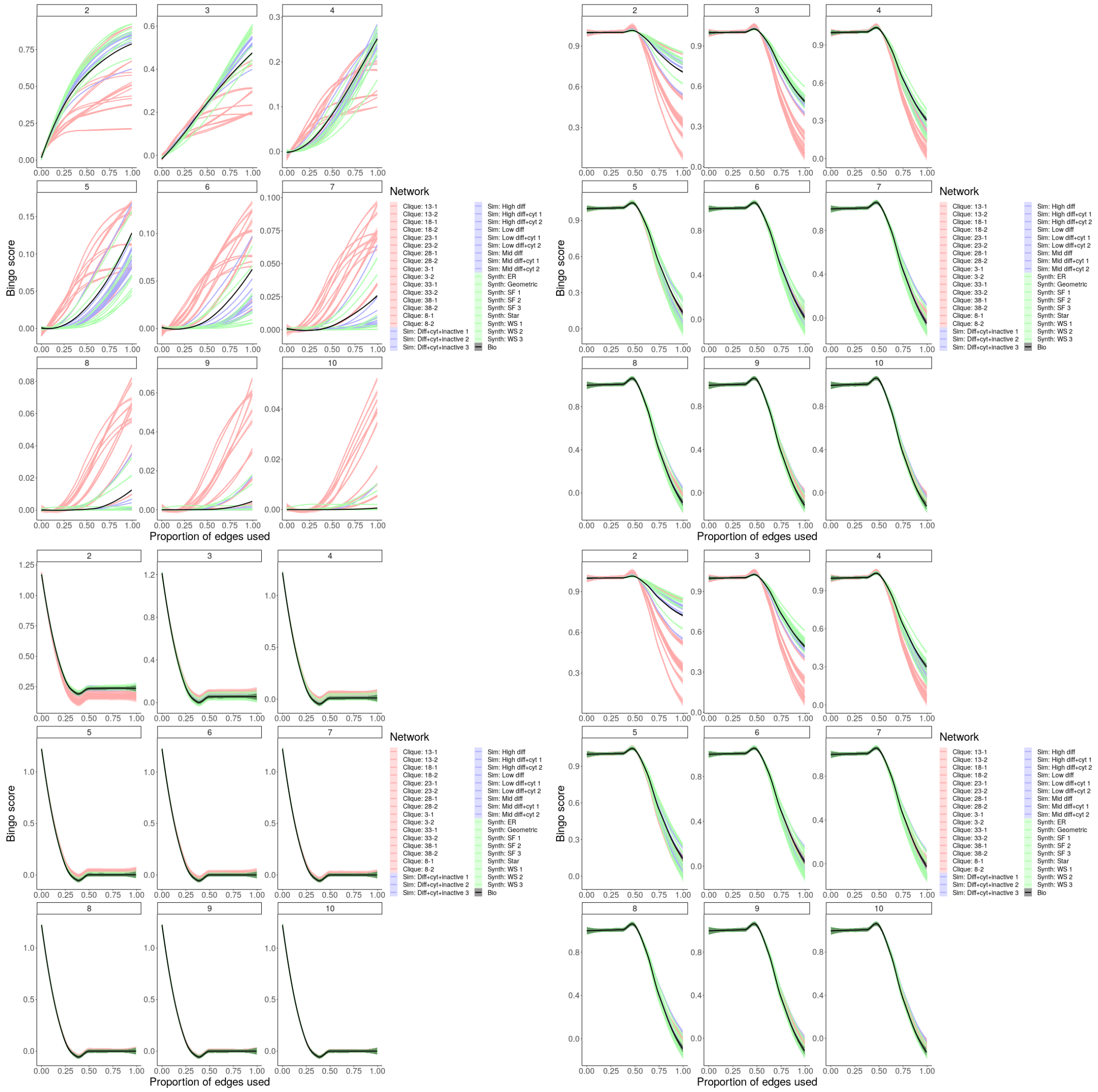

Figure S4: **Mean dynamics of bingo on different networks under different model structures and initial conditions.** (top left) Empty ICs, (top right) full ICs with intermediate degradation, (bottom left) full ICs with high degradation, (bottom right) full ICs with intermediate degradation and low probability of mtDNA exchange. For high degradation cases, no bingos occur for higher values of L.

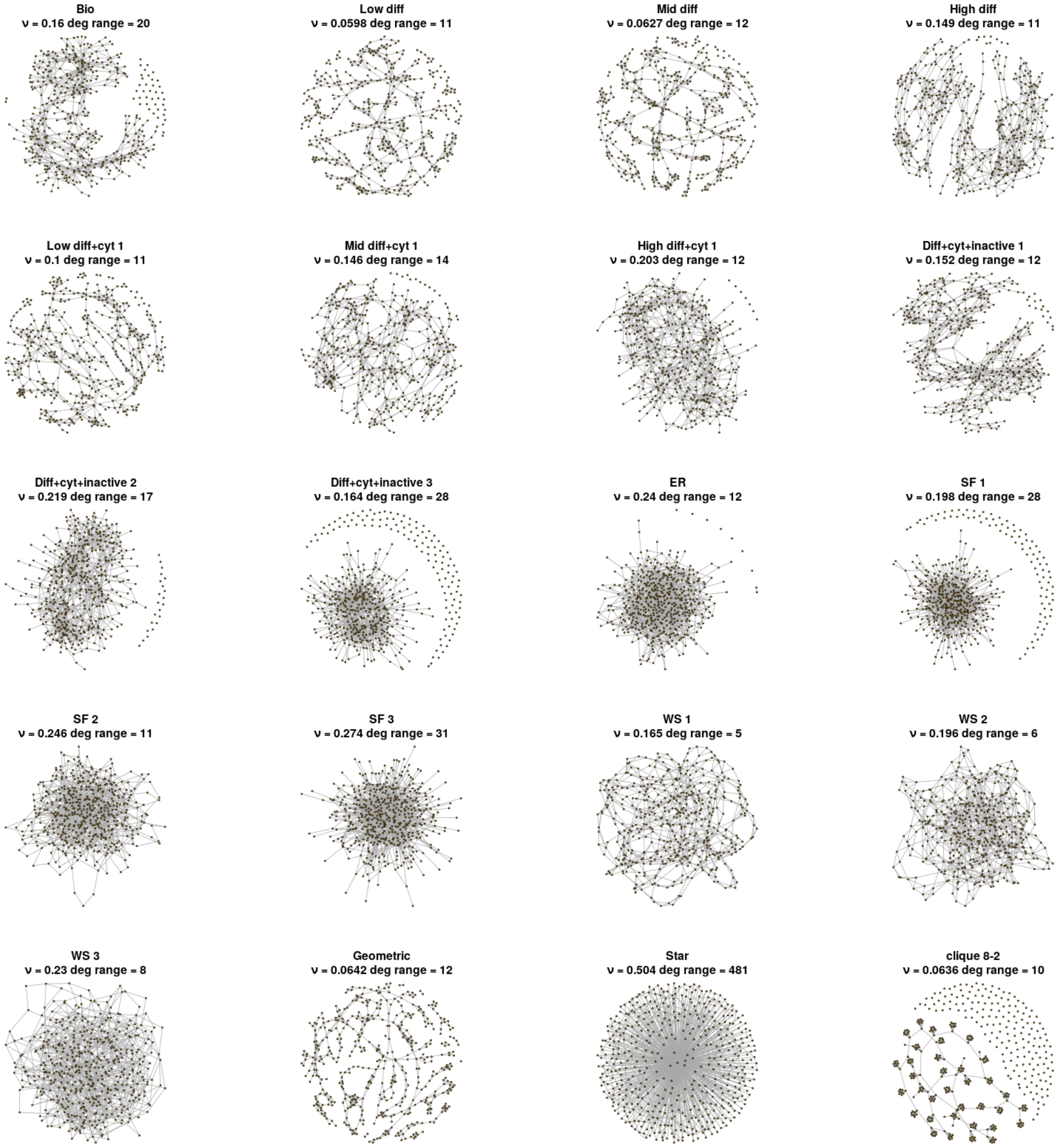

Figure S5: **Comparison of encounter networks from experiment, simulation of mitochondrial dynamics, and general theory.** Visualisations of network structures from the different construction protocols described in Methods, matching (as closely as possible) the statistics of the biological ('Bio') network. One representative 'cliquey' network structure is shown; abbreviations are ER (Erdős-Rényi), SF (scale-free), WS (Watts-Strogatz). Network statistics are ν, global efficiency; and *deg range*, range of degree distribution.

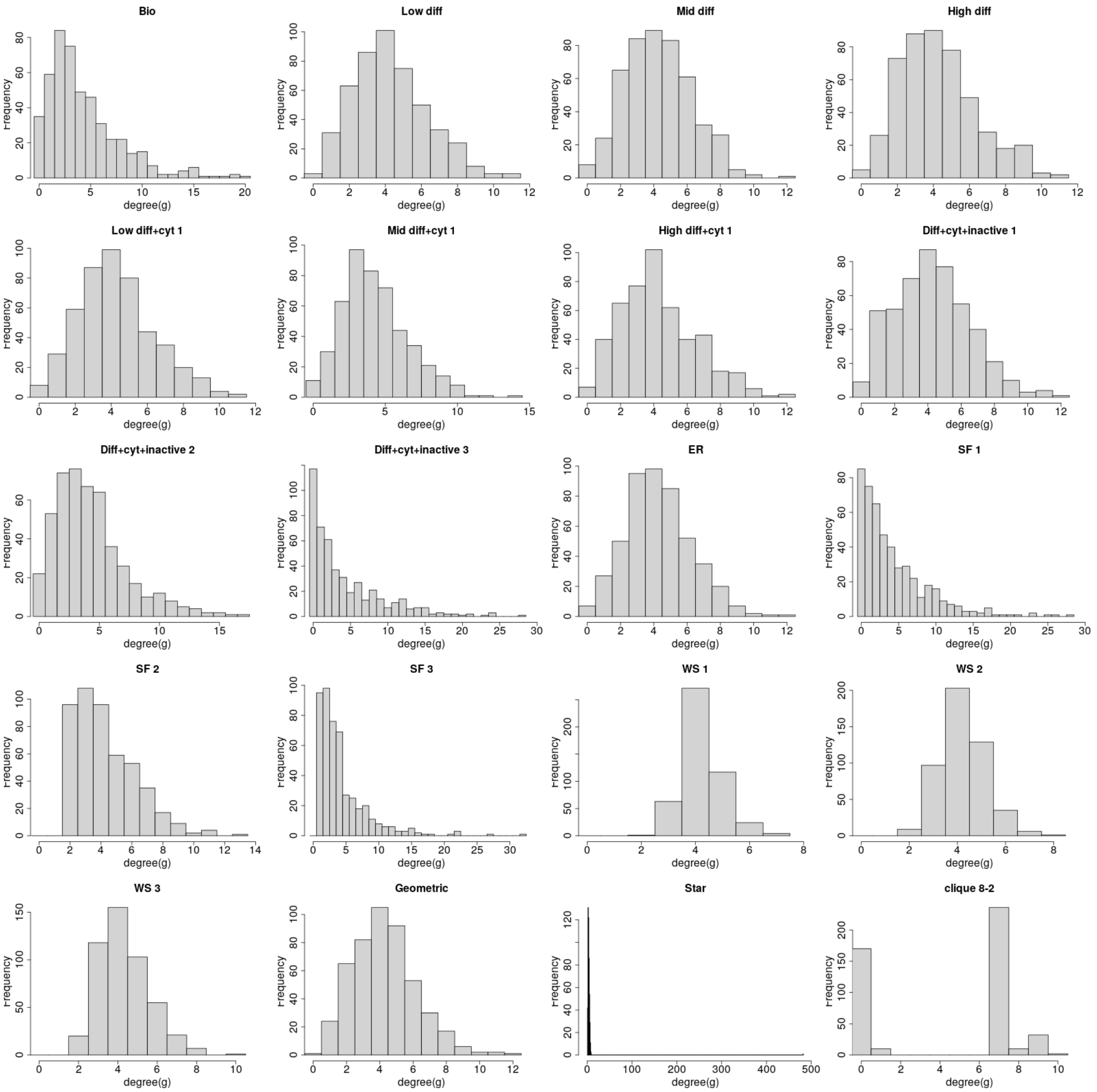

Figure S6: **Degree distributions of encounter networks from experiment, simulation of mitochondrial dynamics, and general theory.** Degree distributions for the ensemble of graphs in Supplementary Fig. 5. One representative ‘cliquey’ network structure is shown; abbreviations are ER (Erdős-Rényi), SF (scale-free), WS (Watts-Strogatz).

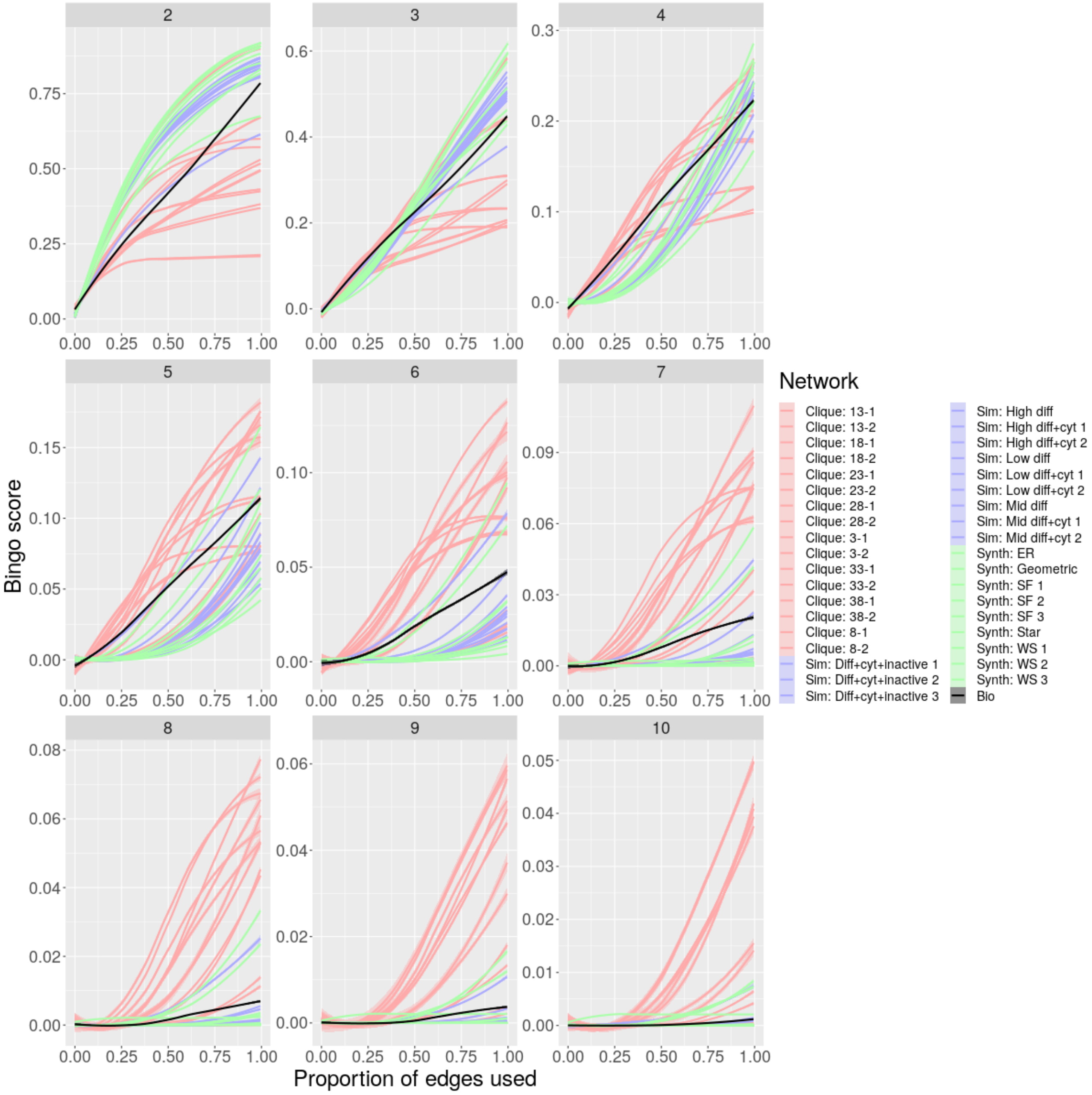

Figure S7: **Bingo dynamics on different networks for different** L**.** Behaviour of bingo score p with proportion of edges q used for genetic exchange, arranged for a range of synthetic networks and their biological partner, for different L. Traces are coloured by the general class of synthetic network. Traces are LOESS fits to $n = 10$ simulations for each case.

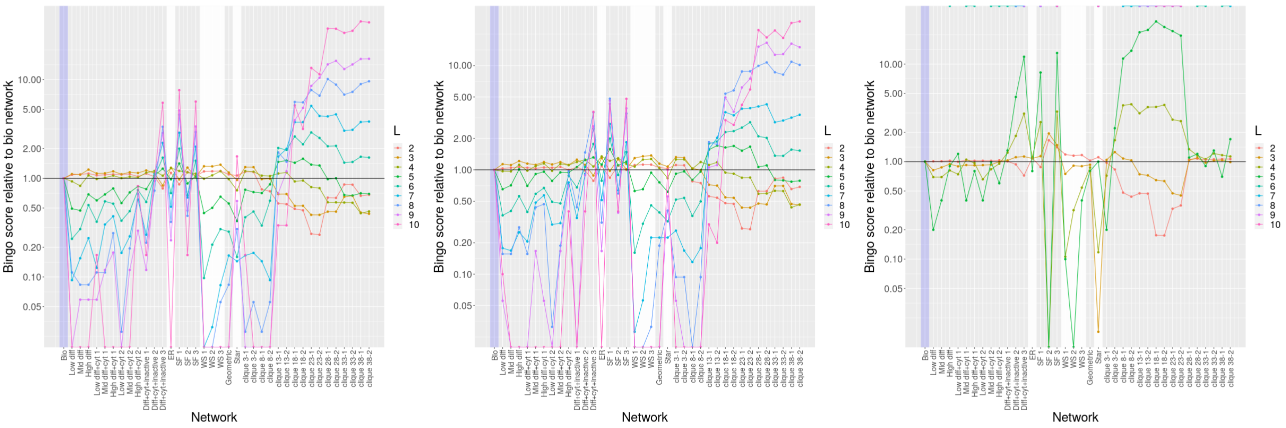

Figure S8: **Comparison of behaviour in different circumstances.** Bingo performance for a number of changes to the experimental setup. Left, normal; centre, singletons removed from biological encounter network; right, biological trajectories pruned to a maximum length of ten frames (23 seconds).

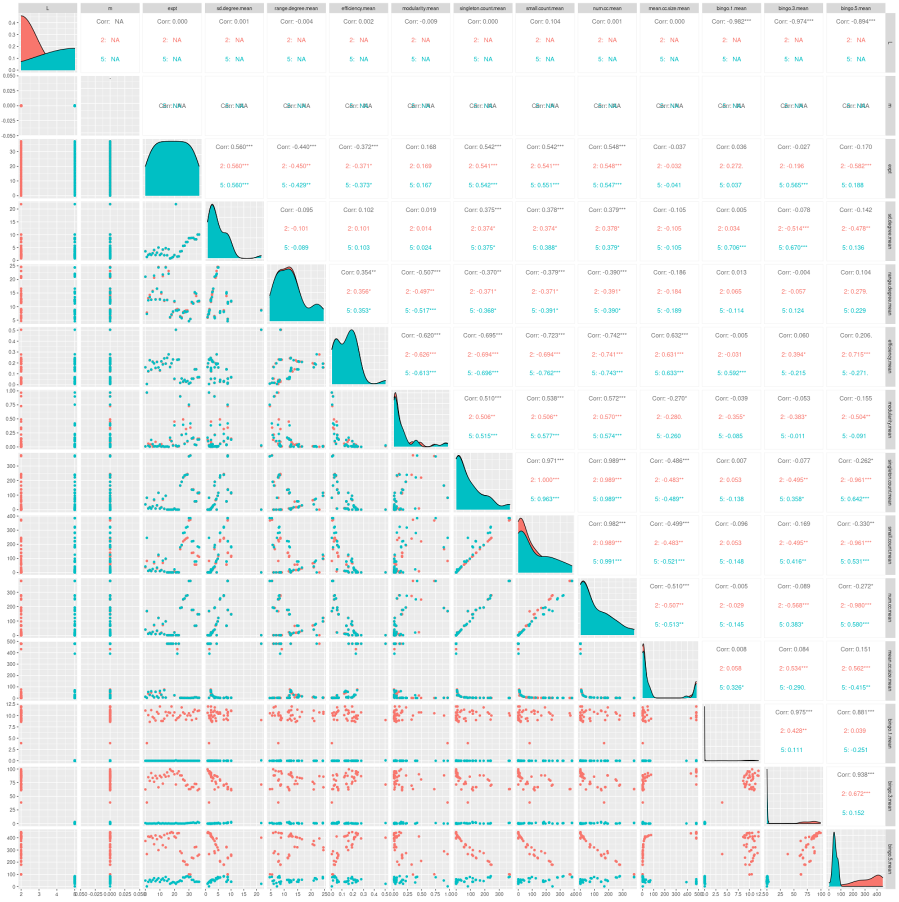

Figure S9: **Network statistics and bingo performance.** Correlations between network statistics and bingo performance, for $L = 2$ (red) and $L = 5$ (blue), with scatter plots under the diagonal, Pearson coefficients above the diagonal (*, $p < 0.05$; **, $p < 0.01$; ***, $p < 0.001$), and histograms of the statistic on the diagonal. Each point is a mean value for a different class of network, taken over 10 generated instances. Labels: *sd.degree* and *range.degree*, degree distribution standard deviation and range; *efficiency*, global network efficiency; *modularity*, network modularity measured using the walktrap algorithm [Pons and Latapy, 2006]; *singleton.count*, number of degree-zero nodes; *small.count*, number of components with size $< L$; *num.cc* and *mean.cc.size*, number and mean size of connected components; *bingo.1/3/5*, bingo score when proportion 0.01/0.1/1 of edges are used for genetic exchange. Although some statistics correlate with bingo performance for a given L, little correlation across L values is visible.

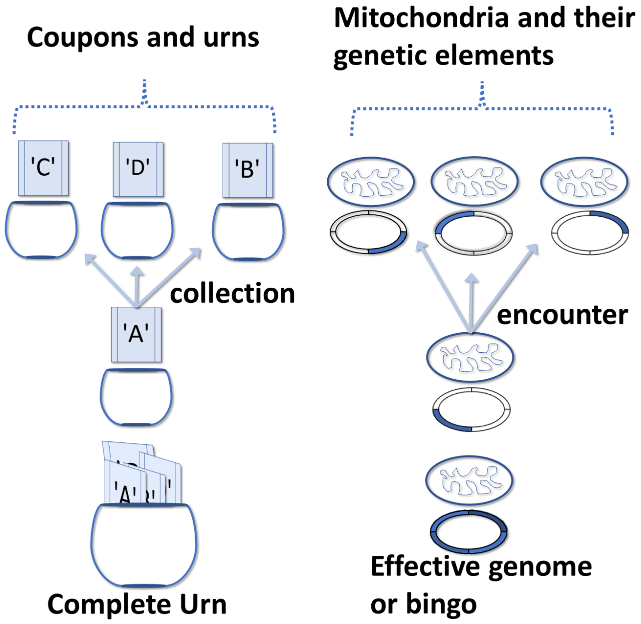

Figure S10: **The analogy between the CCP and bingo.** It is shown and explained the shared terminology between the two concepts and how the coupon collection corresponds to the the assembly of effective genome through encounters with partial genome elements.
